# Asynchronous Space‐Time‐Coding Digital Metasurface

**DOI:** 10.1002/advs.202200106

**Published:** 2022-06-25

**Authors:** Si Ran Wang, Ming Zheng Chen, Jun Chen Ke, Qiang Cheng, Tie Jun Cui

**Affiliations:** ^1^ Institute of Electromagnetic Space Southeast University Nanjing 210096 P. R. China; ^2^ State Key Laboratory of Millimeter Waves Southeast University Nanjing 210096 P. R. China

**Keywords:** asynchronous, automatic spatial scanning of the harmonics, dynamic generation of radar cross sections, frequency discontinuities, space‐time‐coding digital metasurfaces

## Abstract

Recent progress in space‐time‐coding digital metasurface (STCM) manifests itself a powerful tool to engineer the properties of electromagnetic (EM) waves in both space and time domains, and greatly expands its capabilities from the physical manipulation to information processing. However, the current studies on STCM are focused under the synchrony frame, namely, all meta‐atoms follow the same variation frequency. Here, an asynchronous STCM is proposed, where the meta‐atoms are modulated by different time‐coding periods. In the proposed asynchronous STCM, the phase discontinuities on traditional metasurface are replaced with the frequency discontinuities. It is shown that dynamic wavefronts can be automatically realized for both fundamental and high‐order harmonics by elaborately arranging the spatial distribution of meta‐atoms with various time‐coding periods. The physics insight is due to the accumulated rapidly changing phase difference with time, which offers an additional degree of freedom during the wave‐matter interactions. As a proof‐of‐principle example, an asynchronous STCM for automatic spatial scanning and dynamic scattering control is investigated. From the theory, numerical simulations, and experiments, it can be found that the proposed STCM exhibits significant potentials for applications in radars and wireless communications.

## Graphical abstract

An asynchronous space‐time‐coding digital metasurface (ASTCM) is proposed to generate dynamic wavefronts by modulating the meta‐atoms with various periods. It is the first time to introduce frequency discontinuities to the digital coding metasurfaces. The outstanding abilities of the ASTCM include the automatic spatial scanning and dynamic scattering control, thus benefiting its applications in radars and wireless communications.

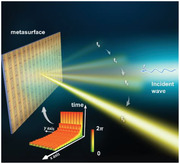

## 1 Introduction

Metasurfaces, as 2D artificial engineering structures^[^ ^1^, ^16^ ^]^ can flexibly and accurately manipulate electromagnetic (EM) waves by introducing the predesigned phase discontinuities among the subwavelength meta‐atoms, along with the advantages including small profile, easy fabrication and lower loss. To simplify the design and optimization process and introduce the information features, in 2014, Cui et al. revisited the metasurfaces from the perspective of digital information and proposed the concept of digital coding metasurfaces,^[^ ^17^ ^]^ where two distinct meta‐atoms with opposite reflection phases are encoded by the digits “0” and “1”, respectively. The phase distributions over the metasurfaces can be represented by the corresponding coding sequences. Furthermore, by integrating active elements like varactors or PIN diodes into the meta‐atoms, the coding states can be switched in real time by changing their biasing voltages. When all coding sequences of the digital metasurface are carefully designed and precalculated,^[^ ^18^, ^19^, ^20^, ^21^ ^]^ it can accomplish various functionalities dynamically in real time with the aid of a field‐programmable gate array (FPGA), yielding the programmable metasurface.^[^ ^17^, ^18^, ^19^, ^20^, ^21^ ^]^

Recently, the concept of space‐time‐coding digital metasurface (STCM)^[^ ^22^ ^]^ was proposed to realize simultaneous controls of harmonic amplitude, phase and polarization by assigning space‐time coding matrix on the metasurface. A wide variety of unique applications such as harmonic beam steering, beam shaping, efficient frequency conversion, nonreciprocal effect, and nonlinear information modulation have been presented, investigated and experimentally demonstrated.^[^ ^23^, ^24^, ^25^, ^26^, ^27^ ^]^ To date, however, all the published STCMs were developed under the synchrony frame. Generally speaking, a digital coding metasurface is said to be synchronous if all meta‐atoms change their states with the same frequency based on a global clock signal. It allows the metasurfaces to modulate the EM properties of the illuminated waves in a rapid and fixed way over a specified length of time. Due to this reason, it is natural to raise an interesting question: Is it possible to create an asynchronous metasurface? What are the key features of the asynchronous metasurface?

In fact, the asynchronous phenomenon is common in our daily life. For example, as the systems in asynchronous relationships have different velocities (or angular velocities and frequencies), relative motions will exist among the systems. For example, near‐earth orbit satellites can spontaneously circle the earth because they are asynchronous with the earth's rotating system. In analogy, in the microwave regime, the similar asynchronous systems are called as frequency diverse arrays (FDAs)^[28^.^37]^ By employing different carrier frequencies across the radiation elements, an FDA can create a dynamic radiation pattern, distinguishing it from the existing phased array. As a special case,^[^ ^37^ ^]^ by applying linear carrier frequencies across the elements, FDA can realize automatic space scanning in a periodic manner without the help of phase shifters used in the phased arrays.^[^ ^38^ ^]^ However, the current FDA is an active radiation system, which fails to manipulate the scattering properties of EM waves as desired.

Recent studies reveal that apart from the interesting and anomalous physics from the phase gradient metasurface, a new class of asynchronous metasurface, namely the frequency gradient metasurface that introduces predefined frequency differences into adjacent unit cells, opens new possibilities to tailor wave–matter interactions, giving rise to the prominent ability of automatic and continuous beam steering over the time.^[^ ^39^ ^]^ However, the reported asynchronous metasurface was still a virtual asynchronous system that employed a normal passive metasurface under the illumination of a frequency‐comb source, which is aided by a silicon metasurface as a beam splitter to provide frequency diverse excitations to individual elements. Such configuration greatly hinders its application in radar or imaging scenarios due to the complicated feeding system. In addition, the passive beam splitter^[^ ^39^ ^]^ prevents the frequency gradient profile of the metasurface from real time changing, and thus making it hard to construct a programmable artificial surface as desired.

Here, we propose an asynchronous space‐time‐coding digital metasurface (ASTCM), and report on its design and experimental characterization. Different from the work in ref. [39] we used the active metasurface, where all coding meta‐atoms are controlled by the external biasing voltages of the embedded varactor diodes. Varied stimulation cycles and coding sequences are chosen to exert programmable frequency gradient of ASTCM, which can remove the need of complicated frequency diverse feeding. The frequency offsets among meta‐atoms provide dynamic phase gradients with the change of time, and realize automatic beam scanning at a specified speed. We also realize dynamic radar cross sections (RCSs) from ASTCM, which upend the conventional notions of the time‐independent RCSs, if the target range and pose remain stable. The proposed ASTCM will spur more applications in the areas of cognized radar and wireless communication in the future.

## 2 Results

### 2.1 Theory

**Figure 1:**
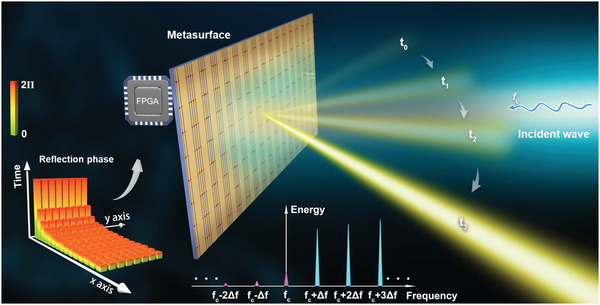
Conceptual illustration of ASTCM. The reflection phase of the meta‐atoms can be dynamically controlled by changing the biasing voltages of the varactor diodes. Coding strategies are displayed in the left‐bottom corner, in which each block represents a coding state. The color of each block represents the periodic reflection phase, and height of each block represents the duration time for one period. The reflection power spectrum is shown at the bottom. In the illustrated example, the time‐coding sequences adopted to the spatial meta‐atoms are similar, whereas the periods are different. When the reflection phase increases linearly and continuously from 0 to 2*π* in different periods, the scattered wave can automatically scan the space with time.

As illustrated in **Figure** **1**, we consider an ASTCM containing **M**×**N** meta‐atoms. The reflectivity of meta‐atoms can be dynamically controlled by applying different voltages to the embedded varactor diodes. In the traditional STCMs,^[^ ^22^ ^]^ all meta‐atoms were modulated with various spatial and temporal coding sequences in the same period. However, for the proposed ASTCM, the modulation period for the meta‐atoms varies a lot at different positions on the metasurface, as shown in the bottom‐left corner of Figure 1, where each block represents the coding states of the spatial meta‐atom, with the color representing the temporal reflection phase, and the height representing the duration time for one modulation period.

According to the previous studies,^[^ ^40^, ^41^ ^]^ the reflectivity, as a periodic function of time, can be expressed as a linear combination of harmonically related complex exponentials. Hence the reflectivity of a meta‐atom in ASTCM can be expressed as

(1) $$\Gamma_{mn}\left(t\right)=\sum_{k=-\infty }^{\infty }a_{mn}^{k}e^{j2\pi k\left(f_{0}+\Delta f_{mn}\right)t}$$

where $a_{mn}^{k}$ represents the reflectivity of the meta‐atom (*m*, *n*) at the *k* ^th^‐order harmonic frequency. It is determined by the temporal coding sequences according to the theory of Fourier Series. *f*_0_ and Δ*f_mn_* correspond to the reference modulation frequency and the additional modulation frequency offset for the meta‐atom (*m*, *n*), respectively. Thus, the actual modulation period of the meta‐atom (*m*, *n*) becomes 1/(*f*_0_+Δ*f_mn_*), which is closely related to the element number. Under the normal incidence of a monochromatic wave at the frequency of *f_c_*, the far‐field scattering pattern of ASTCM can be written as:

(2) $$f\left(t,R,\theta ,\varphi \right)=\sum_{k=-\infty }^{\infty }\sum_{m=1}^{M}\sum_{n=1}^{N}E_{mn}\left(\theta ,\varphi \right)a_{mn}^{k}e^{j\left\{\emptyset_{1}^{k}+\emptyset_{2}^{k}\right\}}$$

where *E_mn_* (*θ*, *φ*) stands for the unit scattering pattern. Here, we suppose that the mutual coupling effects between adjacent meta‐atoms are identical, and the element coefficient *E_mn_* = 1. $a_{mn}^{k}$ accounts for the Fourier coefficient of the *k* ^th^‐order harmonic for the meta‐atom (*m*, *n*). *θ* and *φ* denote the elevation and azimuth angles, respectively. $\emptyset_{1}^{k}$ and $\emptyset_{2}^{k}$ represent the time‐invariant and time‐varying phases, respectively, as can be given by:

(3) $$\left\{\begin{array}{c} \emptyset_{1}^{k}=\frac{2\pi f_{c}}{c}\;d\left(\left(m-1\right)\sin\theta \cos\varphi +\left(n-1\right)\sin\theta \sin\varphi \right)\\ \emptyset_{2}^{k}=2\pi k\Delta f_{mn}\left(t-\frac{R}{c}\right) \end{array}\right.$$

where *d* is the interval between the meta‐atoms in both *x* and *y* directions, and *c* is the speed of light. *R* is the distance from the center of the metasurface to the observation point.

In Equation 3, $\emptyset_{1}^{k}$ is only related to the spatial positions, while $\emptyset_{2}^{k}$ stems from the additional modulation frequency offsets among meta‐atoms. Thanks to such frequency offsets, ASTCM acquires an additional time varying phase $2\pi k\Delta f_{mn}\left(t-\frac{R}{c}\right)$ compared to STCM, making it possible to realize a dynamic harmonic wavefront with the fixed coding sequence. However, for STCM, it relies on the change of coding sequences to implement the fast switch of the wavefront. To better illustrate the features of ASTCM, we consider the following three cases for illustrations.

**Case 1**. The meta‐atoms are modulated with the same time period and the same spatial coding sequences. In this case, the modulation frequency offsets are all equal to zero, and the Fourier coefficients in Equation 2 is irrelevant to the meta‐atom position for the *k* ^th^‐order harmonic, which can be replaced by a complex amplitude *a^k^*. Specifically, the corresponding harmonic scattering pattern can be simplified as:

(4) $$f_{k}\left(\theta ,\varphi \right)=\sum_{m=1}^{M}\sum_{n=1}^{N}a^{k}e^{j\emptyset_{1}^{k}}$$

which is identical to that of the time‐domain coding digital metasurface^[^ ^40^, ^42^ ^]^ As the harmonic amplitudes and phases can be controlled freely by the temporal coding sequences, such metasurfaces can alter the spectral power distributions in a precise manner.

**Case 2**. The meta‐atoms have the same time period but different spatial coding sequences. In this case, ASTCM degenerates into a standard STCM.^[^ ^22^ ^]^ Thus the Fourier coefficients of the meta‐atoms are highly dependent on the element location. The *k* ^th^‐order harmonic scattering pattern becomes:

(5) $$f_{k}\left(\theta ,\varphi \right)=\sum_{m=1}^{M}\sum_{n=1}^{N}a_{mn}^{k}e^{j\emptyset_{1}^{k}}$$

where $a_{mn}^{k}$ is dependent on the location of the element (*m*, *n*). In comparison to Case 1, the metasurface possesses the capability of manipulating the harmonic wavefront and the spectral power distributions simultaneously.

**Case 3**. The meta‐atoms are modulated by different time periods and different spatial coding sequences. In this case, the *k* ^th^‐order harmonic scattering pattern can be expressed as:

(6) $$f_{k}\left(t,R,\theta ,\varphi \right)=\sum_{m=1}^{M}\sum_{n=1}^{N}a_{mn}^{k}e^{j\left\{\emptyset_{1}^{k}+\emptyset_{2}^{k}\right\}}$$

Distinguished from Case 1 and Case 2, $\emptyset_{2}^{k}$ is non‐zero here. Consider two neighboring meta‐atoms with the element numbers (*m, n*) and (*p, q*), the phase gradient between the two elements is (*ϕ*_*mn*_‐*ϕ*_*pq*_)/*d_e_ =* $2\pi k\left(\Delta f_{mn}-\Delta f_{pq}\right)\left(t-\frac{R}{c}\right)/d_{e}$, in which *d_e_* represents the element spacing, and the propagation phase difference from the observation point to the two individual element centers are neglected for better discussion. In fact, once the frequency offsets Δ*f_mn_* and Δ*f_pq_* are fixed, the phase gradient is a function of time due to the introduction of $\emptyset_{2}^{k}$, implying that the harmonic wavefronts will change automatically over the time according to the generalized Snell's law. This distinct feature of ASTCM is especially valuable for typical applications such as automatic spatial harmonic‐beam scanning and scattering suppression, as will be discussed below.

For 1D harmonic beam scanning, Equation 6 can be further reduced to:

(7) $$f_{k}\left(t,R,\theta \right)=\sum_{n=1}^{N}a_{n}^{k}\;e^{j2\pi \left(n-1\right)\left\{\frac{f_{c}d}{c}\sin\theta +k\Delta f\left(t-\frac{R}{c}\right)\right\}}$$

where Δ*f* accounts for the modulation frequency offset between adjacent columns. Moreover, the scanning period is decided by 1/Δ*f*. In Equation 7, the initial beam direction can be altered by $a_{n}^{k}$ or the time varying phase $k\Delta f(t-\frac{R}{c})$. For simplicity, we set the starting observation time as *t* = *R*/*c* so that the time‐varying phase becomes zero. Then we can control the initial beam direction by simply changing $a_{n}^{k}$. Additionally, according to Equation 7, the scanning speed of the harmonic beam can be derived as:

(8) $$d\theta /dt=-kc\Delta f/f_{c}d\cos\theta$$

The above equation indicates that by increasing or decreasing Δ*f*, it is possible to find an excellent balance between the scanning speed and scanning range per unit time. More specifically, we can use small Δ*f* (low speed) in the angular range of particular interest in order to acquire more details of the target, while large Δ*f* is employed for rough detection in the remaining regions. Also, by changing the sign of Δ*f* one can control the scanning direction of the patterns.

However, if distinguished frequency gradient distributions are introduced by controlling the frequency offsets among columns, the reflected energy from ASTCM will be dispersed into more frequency components compared to STCM, and thereby achieves the scattering suppression. More importantly, RCS of ASTCM turns to be dynamic when the metasurface keeps still. Under the normal incidence of a plane wave, the backward RCS reduction of ASTCM compared to a metallic plate with the same size can be calculated as:^[^ ^17^ ^]^

(9) $$\sigma_{R}=20\log\left|M\times N/f\left(t,R,0,0\right)\right|$$

where *f*(*t*, *R*, 0, 0) stands for the scattering pattern of ASTCM in Equation 2 at *θ* = 0 and *ϕ* = 0. Since *f*(*t*, *R*, 0, 0) is a function of the time, *σ*_*R*_ becomes a time‐dependent variable under this condition. Besides, from Equation 2 and 9, one can alter RCS at will based on the well‐designed modulation period distributions on the metasurface.

Then we proceed to investigate the realization of modulation frequency offset in our design. As discussed in Equation 1, the metasurface is driven by a monochromatic wave instead of a multi‐frequency signal as done in ref. [39]. However, from the construction of STCM, it is easy to find that all meta‐atoms are controlled by the same period, that is usually an integer multiple of the machine cycle from a global clock signal. In other words, the trigger signals of the I/O ports that supply external biasing voltages to the diodes in meta‐atoms are identical, and thus allowing synchronization in the refresh of phase states for all elements. Similarly, if various cycles are elaborately employed for the internal meta‐atoms by FPGA, it is possible to create different periods during the modulation process. Therefore, the temporal coding period enables the nonlinear frequency conversion from the incident frequency *f_c_* to *f_c_* + Δ*f_mn_*, and the reflected wave from each meta‐atom will have the frequency offset as a result. Since in each tiny machine cycle we can still change the diode biasing voltages freely, the spatial coding sequences for meta‐atoms can be altered inside different modulation periods to satisfy the goal of asynchronous space‐time coding as desired.

From Equation 6, dynamic harmonic scattering patterns can be efficiently synthesized by $a_{mn}^{k}$ (depending on the coding sequence from Equation 5) and the modulation frequency offset Δ*f_mn_*, and hence it is convenient to implement automatic beam scanning for arbitrary‐order harmonics. Furthermore, the extraordinary scattering fields of ASTCM lead to time‐varying RCSs, which has never been encountered for natural objects. We will address the two issues thereinafter in more details.

### 2.2 Automatic Space Scanning of Harmonic Scattering Patterns

Unlike the traditional FDA that enables automatic beam scanning for the carrier wave^[^ ^28^, ^37^ ^]^ the proposed ASTCM can realize efficiently nonlinear frequency conversions from the fundamental frequency to high‐order harmonics, and therefore realize automatic scanning wavefronts for any high‐order harmonics at will.^[^ ^40^, ^41^ ^]^ For instance, to ensure strong interferences from the reflected first‐order harmonic waves from the meta‐atoms, we should first consider a temporal coding sequence to implement the spatial mixing effect.^[^ ^42^ ^]^ Specifically, the reflectivity of a column on the metasurface can be expressed as:

(10) $$\Gamma \left(t\right)=A\left(t\right)e^{j\varphi \left(t\right)}$$

where *A*(*t*) and *ϕ*(*t*) respectively represent the amplitude and phase of the reflectivity. We assume that the amplitude is uniform, whereas the phase is a periodic function of time:

(11) $$\varphi \left(t\right)=p\times \mathrm{mod}\left(t,T\right)$$

in which **mod** (*t*, *T*) denotes the remainder of *t*/*T*, and *p* is the phase slope. According to ref. [43], when *pT* = 2*mπ* (m = ± 1, ± 2, …), the complex Fourier coefficient of the *k*th‐order harmonic can be obtained as:

(12) $$a_{k}=\left\{\begin{array}{cc} 1, & m=k\\ 0, & m\neq k \end{array}\right.$$

Equation 12 indicates that if *pT* is integral multiple of 2*mπ*, only the *m*th‐order harmonic can survive in the reflected wave from that column. Therefore, we can get a highly purified 1^st^ order harmonic with the frequency shift Δ*f* = 1/*T* with *m* = 1. Accordingly, for a metasurface with *N* columns, if we use 1/Δ*f*, 1/2Δ*f*, ⋅⋅⋅, 1/*N*Δ*f* during phase modulations for column *1* ^#^, *2* ^#^, *3* ^#^, ⋅⋅⋅, *N* ^#^, we will get the frequency‐diverse reflected waves with the frequency offset of Δ*f*, 2Δ*f*, ⋅⋅⋅, *N*Δ*f* from the incident frequency. In the left‐bottom corner of Figure 1, we show the temporal reflection phase distribution of meta‐atoms in whole ASTCM, where various space‐time‐coding matrices should be applied to the digital elements as the modulation waveform. The modulation period varies dramatically among the columns in the *x* direction, while it remains unchanged in the *y* direction. Since the modulation frequency is increased monotonically, the modulation period decreases correspondingly as the column number goes up.

**Figure 2:**
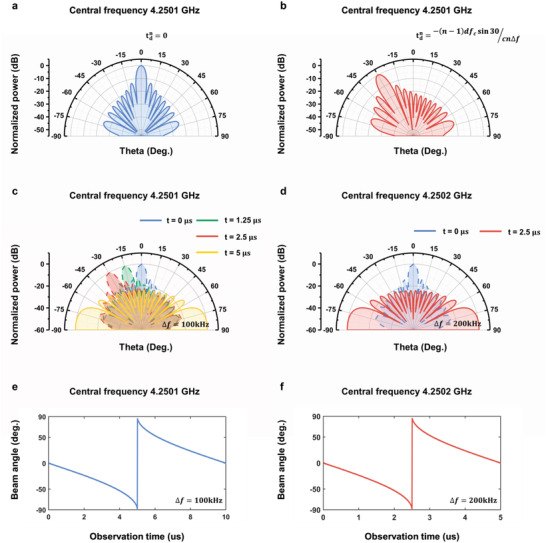
Time‐dependent scattering patterns of the first‐order harmonic. a,b) The initial scattering patterns at *t* = 0. The initial beam direction is adjusted by introducing specific time delay ($t_{d}^{n}$) to each column. c,d) The change of patterns as *t* grows, in which Δ*f* = 100 kHz and 200 kHz, respectively. e,f) Dependence of the beam angle on the observation time when Δ*f* = 100 kHz and 200 kHz, respectively.

For the first‐order harmonic, we investigate two fundamental parameters: the starting direction and scanning speed in the automatic beam steering process. To adjust the starting angle of the harmonic beam, we introduce different time delays into the reflectivity functions of all columns, so that the initial phase distributions on the metasurface can be effectively controlled without affecting the harmonics intensity distributions^[^ ^23^, ^43^ ^]^ According to the theory of Fourier Series, the reflectivity with a time delay *t_d_* will lead to an additional phase 2*πkf_o_t_d_* with the amplitude unchanged for the *k* ^th^ ‐order harmonic. Note that the temporal reflection phase that follows Equation 11 with *m* = 1 is employed to convert the energy from the incident frequency to the 1^st^‐order harmonic frequency. Here we set the parameters *N*, *d, f_c_*, and Δ*f* as 16, 35 mm, 4.25 GHz, and 100 kHz, respectively (see Equation 7). In addition, the far‐field observation distance and initial observation time are *R*_0_ and $R_{0}/c$. **Figure** **2**a shows the initial beam direction at 0^○^ without any time delay across the columns, while Figure 2b shows that the initial beam direction is steered to −30^○^ after some specific time delays are introduced to the columns. Specifically, $t_{d}^{n}$ denotes the time delay introduced to the *n* ^th^ column, which will add an extra phase delay of $-2\pi \Delta ft_{d}^{n}$ to the first‐order harmonic,^[^ ^43^ ^]^ and *t* is the observation time.

To adjust the scanning speed of the harmonic beam, we set Δ*f* = 100 and 200 kHz, respectively, and the remaining parameters are kept the same as those in Figure 2a. Specifically, the centeral frequencies of the patterns are 4.2501 and 4.2502 GHz respectively, with Δ*f* = 100 and 200 kHz. The scanning pattern of the 1^st^‐order harmonic at different time are plotted in Figure 2c,d. Moreover, the beam angles over time are shown in Figures 2e,f. We can find that it takes 5 us to complete the scan of a quarter of space when Δ*f* = 100 kHz, while the total scanning time is cut by half (i.e., 2.5 us) if Δ*f* is further increased to 200 kHz. Therefore, the offset frequency Δ*f* offers an efficient way to regulate the scanning speed of the harmonic scattering pattern.

As the starting angle and the scanning time of the harmonic beams can be independently controlled by the time delay and the additional frequency offset, one can flexibly realize automatic scanning for any specific area by ASTCM.

### 2.3 Dynamic RCS Controls of ASTCM

From Equation 2, we find that the scattering patterns of ASTCM are dependent on the operation time and the observation distance simultaneously, which differs greatly from the scattering features of the traditional objects. Once ASTCM is located on the surface of a static target, it will lead to time and distance varying RCSs. While the traditional RCS is usually a time‐independent term for the natural objects.

**Figure 3:**
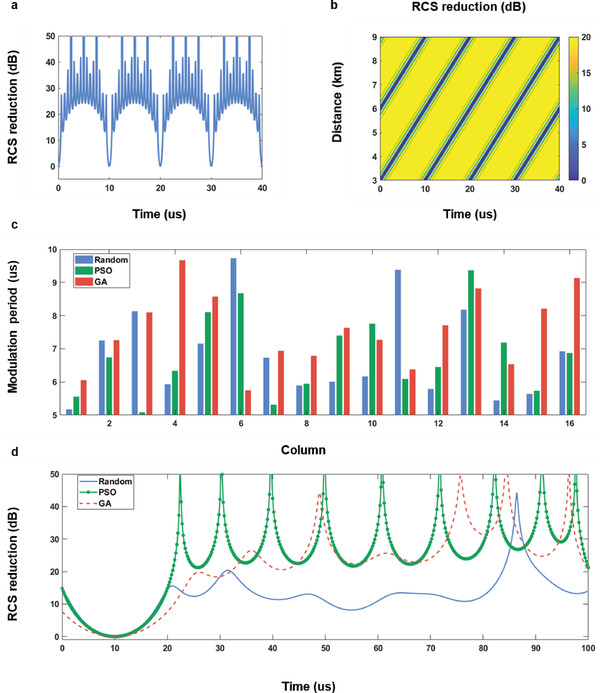
a) The dependence of RCS reduction on the observation time at the distance of 3 km. b) The dependence of RCS reduction on the observation time and distance. c) The distributions of the modulation periods at different columns acquired through the random selection, and optimization algorithms—PSO, GA. d) The corresponding RCS reduction curves with the time at 3 km.

We calculate the backward RCS reduction of an ASTCM consisting of 16 columns and 8 rows based on Equation 9. The coding strategy is the same as that in Figure 1 without additional time delays across the metasurface. Here Δ*f* and *f_c_* are 100 kHz and 4.25 GHz, respectively. The observation distance is set from 3 to 9 km. The dependence of the backscattered RCS reduction on the time and distance is illustrated in **Figure** **3**a,b. At the distance of 3 km, the RCS reduction is over 20 dB in most of the time, except that at some moments it reduces to zero in Figure 3a, because there is no RCS reduction when $\emptyset_{2}^{k}$ is the integral multiple of 2*π* from Equations 2 and 9. Also, it can be found that the RCS reduction effect is dramatic in most areas, but with periodic fringes of nearly 3 km at each moment.

Owing to the low RCS and dynamic features, ASTCM can serve as a good candidate for the stealth or camouflage technologies. But the periodic RCS reduction curves in both time and space domains makes it unfavorable for real applications. A simple recipe to overcome this challenge is to adjust the frequency interval between neighboring columns. For instance, the modulation frequencies across the columns can be randomly selected in the range from 100 to 200 kHz, while other parameters are identical to those in Figure 3a.

The random distribution of modulation frequencies leads to random frequency gradients across the whole metasurface, which in turn breaks the undesired temporal and spatial regularity. The modulation period distribution and the corresponding RCS reduction are presented in blue in Figure 3c,d, respectively, in which we can see that the periodicity disappears as expected. To further reduce RCS of ASTCM, two optimization algorithms, Genetic Algorithm (GA) and Particle Swarm Optimization (PSO) are employed to generate two sets of modulation periods across the columns, as shown in red and green respectively in Figure 3c. The RCS reductions given in corresponding color in Figure 3d are approximately greater than 20 dB after 30 µs.

We remark that the RCS reduction phenomenon is originated from the interference of EM waves, which is reflected from the columns with different modulation frequencies. In fact, this is actually an equivalent approach to lower RCS and deceive the radar with a certain bandwidth, because if the detector operates at a single frequency, such mechanism is no longer valid at that time.

## 3 Experimental Section

**Figure 4:**
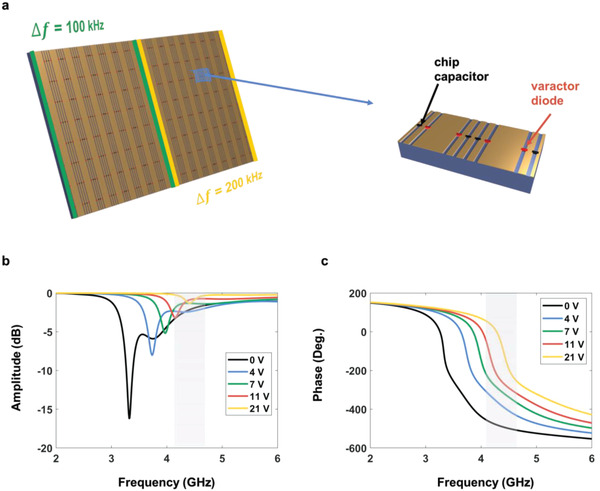
Structure of the metasurface, one of its meta‐atoms zoomed in, and the reflectivity of one meta‐atom. a) Metasurface is divided into two parts for modulating with different temporal coding periods. Each meta‐atom on the metasurface contains four chip capacitors and four varactor diodes. b,c) The reflection amplitude and phase of the meta‐atom.

To validate the dynamic properties of ASTCM experimentally, an existing prototype made of 16×8 meta‐atoms was employed for experiments, with the total dimension of 384×126 mm^2^. The prototype was used for harmonic generation in refs. [23, 43, 44], with the element details shown in **Figure** **4**a. The meta‐atom comprised three layers. The top and bottom layers were made of copper, whereas the middle dielectric layer was F4B (*ε*_*r*_ = 3.0, tan *δ* = 0.0015). Each meta‐atom contained four chip capacitors and four varactor diodes. The varactor diodes were controlled by using biasing voltages to alter the reflection phases of the meta‐atom rapidly. Full‐wave simulations of the meta‐atom were performed with the commercial software package, CST Microwave Studio 2016. The reflection amplitude and phase of the meta‐atom under different bias voltages are presented in Figure 4b,c, respectively. It was found that the reflection phase range was beyond 2*π* near the frequency 4.25 GHz, and the amplitude fluctuation was less than 3 dB. Moreover, on the metasurface, each column shared the same control signal. The aperture efficiency of a meta‐column was 77%, which was obtained by calculating the monostatic RCS reduction relative to a metallic plate with the same size under the normal incidence.^[^ ^17^ ^]^

Two experiments were carried out. The first one was to test RCSs of the metasurface to inspect the predicted time varying scattering pattern. The second one was to validate the automatic spatial scanning of harmonic patterns. During the first experiment, the whole metasurface was divided into two parts, while the left and right ones were modulated with different periods. The same coding sequence was applied to the two regions.

**Figure 5:**
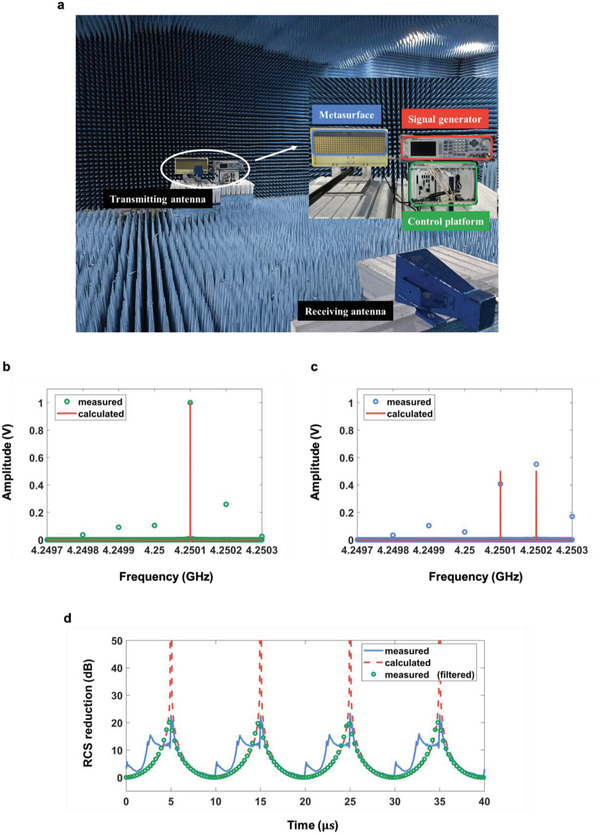
Measurement setup in an anechoic chamber and the measurements. a) The metasurface is illuminated by the EM waves from a transmitting horn antenna connected by the signal generator, and a receiving antenna is used to receive the backscattered EM waves. b,c) Normalized amplitudes corresponding to different frequency modulations in the backscattered direction. b) The entire metasurface is modulated with the period of 10 µs. c) The left and right parts of the metasurface are modulated with different time‐coding periods, and their modulation frequency offset is 100 kHz. d) Comparison of the measured and calculated RCS reductions of the proposed ASTCM.

The first experiment was conducted in a standard microwave anechoic chamber, with the measurement configuration shown in **Figure** **5**a. The metasurface was illuminated by a linearly polarized horn antenna connected to a microwave signal generator (Keysight E8267D) at the frequency of 4.25 GHz. Meanwhile, another horn antenna was fixed in the far field region to receive the backscattered EM waves. To generate periodic time‐varying reflection coefficients, a commercial platform (PXIe‐1082, NI Corp.) that consisted a high‐speed I/O bus controller, an FPGA module, a digital‐analog conversion module, a DC power supply module, and a timing module was used.^[^ ^42^, ^43^ ^]^ According to the mapping relationship between the reflection phase and the biasing voltage, it was easy to generate monotonically increased phase responses in different modulation periods, as required in Figure 1.

To observe the dynamic scattering phenomena in experiment, first the nonlinear harmonic generation was tested with the temporal coding strategy, as shown in Figure 1, and then the dynamic RCS reductions of the proposed ASTCM compared to a control metallic plate with the same size were monitored. For validating the nonlinear harmonic generation, the whole metasurface was modulated to convert the carrier frequency to the first‐order harmonic frequency. The right and the left parts of the metasurface have the same modulation frequency of 100 kHz. The normalized harmonic amplitude distributions is illustrated in Figure 5b. From the comparison between the calculated and measured results, it was clearly noted that most of the incident energy was converted from the carrier wave at *f_c_* = 4.25 GHz to the first‐order harmonic at 4.2501 GHz. Specifically, the conversion efficiency was ≈66.74%. Besides, the energy conversion to the unwanted high‐order harmonics mainly stems from the amplitude fluctuation during the phase modulation. Then the temporal coding sequences were kept unchanged, and the modulation frequencies were set to 100 kHz for the left part (columns 1^#^–8^#^) and 200 kHz for the right part (columns 9^#^–16^#^), respectively, as illustrated in Figure 4a. Hence the additional modulation frequency offset between the two parts was 100 kHz. The normalized harmonic amplitude spectra of measured scattered field are shown in Figure 5c. It is clearly shown that the measured results have good agreement to the theoretical calculations, in which the first and second order harmonics were apparently dominant. However, it was also noted that the amplitudes for the desired 1^st^ and 2^nd^order harmonics are unequal, and the fundamental, the higher‐order harmonics are still not totally removed, which is slightly different from theoretical hypotheses. The emergence of the fundamental and higher‐order harmonics can be ascribed to the amplitude fluctuation during the phase modulation and fabrication error of the sample.

Figure 5d showed the RCS reductions of the sample from 0 to 40 µs. It was seen that the echo signal followed the same trend with the increase of time, which was a periodic function since the modulation frequency is linearly varied across the spatial parts. However, the measured curve was not so smooth because there are some higher‐order harmonic components in the scattering spectrum during the nonlinear frequency conversion process, as indicated in Figure 5b. To validate our analysis, a band‐pass filtering operation wass applied to the measured signal, with the lower and higher cutoff frequencies of 4.25 GHz + 50 kHz and 4.25 GHz  + 250 kHz, respectively. The filtered result agrees with the theoretical calculations very well, indicating that the undesired‐order harmonics still play an important role in the backscattering waves. Furthermore, the nulls in the measured curve is not as deep as expected, primarily due to the unequal amplitudes of the two frequency components, as shown in Figure 5c, which can be improved when the ASTCM performance wass further optimized in the future.

**Figure 6:**
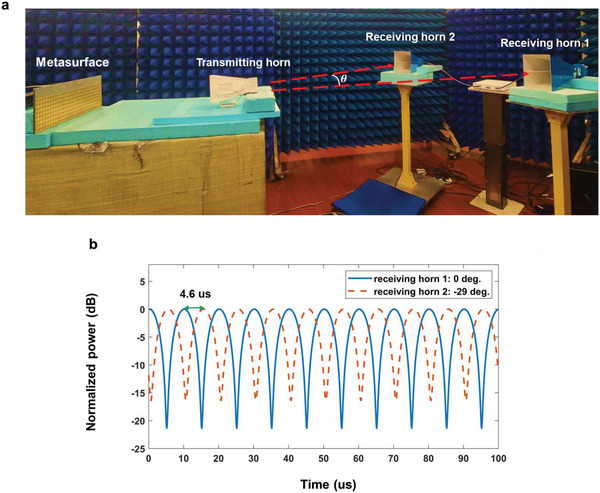
Validation of automatic spatial scanning of harmonic patterns. a) Measurement setup in an in‐door scenario. The reflection signals from the metasurface under the excitation of a transmitting horn antenna are acquired by two recieving horn antennas at the same distance and different directions. b) The measured signal powers received by the two horns over time.

The second experiment was conducted in an in‐door scenario, as shown in **Figure** **6**a. Because no high‐speed turntable and spectrum analyzer were present to measure the scattering pattern of fast scanning beam, an indirect method was used to prove the auto‐scanning property of ASTCM as widely used in the frequency diverse array (FDA) radars. During the experiment, the metasurface wass excited by a transmitting horn in front of it. The reflected waves from the metasurface were simultaneously received by two horns at the same distance but different directions. Due to the auto‐scanning property of ASTCM, there wass a lag time between the signals received by two horns. Assuming that the angle between two receiving horn antennas is *θ*, from Equation 7, the lag time is − (*f_c_d*/cΔ*f*)sin *θ*, where *d* is the interval of the adjacent regions with different modulation manners.To better observe the lag time, the tag time was chosen as 1/2Δ*f*. Also, the modulation frequency (100 kHz) and carrier frequency (4.25 GHz) were the same as those in the previous experiment. Moreover, every adjacent three columns of the metasurface share the same modulation signal, so that *d*  = 72 mm. Hence, the lag time was 5 µs, and the angle between two horns was −29.35°. The undesired harmonics are filtered out from the temporal signals received by the two horns, and the filtered signal powers over time are illustrated in Figure 6b. The lag time of the two signals was 4.6 µs, which was close to the predicted value of 5 µs. The small deviation may come from the slight position error of the two horns in the experiment, since from Equation (7) the time difference of 0.4 µs corresponded to an angle difference of 2.55°. However, the auto‐scanning property can be clearly observed from the measurement results.

## 4 Conclusions

We proposed an asynchronous version of the SCTM, ASTCM, for dynamic wavefront manipulations. Distinct from the reported synchronous coding digital metasurfaces, it offers new degrees of freedom by using different modulation periods for the composing meta‐atoms, which lead to the time‐varying interference scattering patterns with automatic time scanning property and dynamic RCS controls that have never been exhibited by the traditional materials. To confirm the validity of our theory, we fabricated a sample and tested its RCS controls within a short time period and the auto‐scanning property. The experimental results are consistent with the theoretical predictions, proving the unique property of the proposed ASTCM and suggesting a possible route to new radar and wireless communication technologies.

## Conflict of Interest

The authors declare no conflict of interest.

## Data Availability

The data that support the findings of this study are available in the supplementary material of this article.
